# Into the void: ECM fungal communities involved in the succession from rockroses to oak stands

**DOI:** 10.1038/s41598-023-37107-y

**Published:** 2023-06-21

**Authors:** Ignacio Sanz-Benito, Tim Stadler, Olaya Mediavilla, María Hernández-Rodríguez, Juan Andrés Oria-de-Rueda, Tatek Dejene, József Geml, Pablo Martín-Pinto

**Affiliations:** 1 Sustainable Forest Management Research Institute, University of Valladolid, Avda. Madrid 44, 34071 Palencia, Spain; 2 University for Sustainable Development Eberswalde, Schickler Street 5, 16225 Eberswalde, Germany; 3 IDForest-Biotecnología Forestal Aplicada, Calle Curtidores 17, 34004 Palencia, Spain; 4 Central Ethiopia Environment and Forestry Research Center, P.O. Box 30708, Addis Ababa, Ethiopia; 5 ELKH-EKKE Lendület Environmental Microbiome Research Group, Eszterházy Károly Catholic University, Leányka U. 6, 3300 Eger, Hungary

**Keywords:** Forestry, Forest ecology

## Abstract

Oak forests accompanied by *Cistus* species are a common landscape in the Mediterranean basin. It is argued that *Cistus* dominated fields serve as recruitment areas for *Quercus* seedlings, as they help in the transmission of the fungal community through vegetative succession in these ecosystems. To test these assumptions, we analyzed the fungal community in terms of its richness and composition, taking into account the effects of host (*Oaks* vs. *Cistus*) and forest structure, mainly based on age. Edaphic variables related to the different structures were also analyzed to examine how they evolve through succession and relate to shifts in the fungal community. No differences in fungal richness were observed between old *Cistus* stands and younger *Quercus*, while a brief increase in ECM richness was observed. Community composition also showed a greater overlap between old *Cistus* and young *Quercus* stands. We suggest that the most important step in fungal transfer from one host to another is the shift from the oldest *Cistus* fields to the youngest *Quercus* stands, with the genera *Amanita*, *Cortinarius*, *Lactarius*, *Inocybe*, *Russula*, and *Tomentella* probably playing a major role. In summary, our work has also revealed the network of fungal community structure in the succession of *Cistus* to Oak stands, it would suggest that the fungi share niches and significantly enhance the ecological setting of the transition from *Cistus* to *Oak* stands.

## Introduction

The Mediterranean basin is an area that has been greatly altered due to different kinds of disturbances and dynamics, the most relevant of which at present are recurrent wildfires and land abandonment^1–3^. As a result of these disturbances, secondary succession processes are widespread, with the pre-existing vegetation being replaced by a rapidly colonizing woody understory^4^. Different studies have focused on the effect that this land-covering dynamic via shrubs is having on distinct ecological processes, such as tree seedling recruitment^5,6^, soil quality^7,8^, disturbance-adapted drifts in vegetation^9^ and carbon stock shifts^10^. With regard to the entangled life of the soil, a number of studies have investigated the effects of these alterations on the fungal community^11–14^, as well how mycorrhizal processes promote seedling survival under harsh conditions^15,16^. Moreover, it has been observed how already mycorrhizal developed shrubs helps in the tree seedling establishment through mycorrhizal transference^17^. Although few studies have examined how the fungal community accompanies this successional process^18^, it has been seen how ECM fungi community could become a key driver of secondary succession^19^. As well, it has been observed that arbuscular mycorrhizal fungi also lead the plant community composition and diversity through succession^20^, creating an interesting researching niche in Mediterranean ecosystems.

*Cistus* is a widely dispersed plant genus in the Mediterranean area that is found in extensive shrublands, mainly in the Iberian Peninsula. *Cistus ladanifer* is the dominant shrub in acidic oligotrophic soil; however, it is also able to tolerate calcareous lands^21^, and soils with nutrient imbalances and unfavorable conditions^22^, making it a highly successful recolonizing species after a disturbance. Although the seeds have the capacity to germinate under a wide range of conditions, seeds can remain dormant enabling *C. ladanifer* to deposit a huge seeds soil bank^23^. Given that *C. ladanifer* is usually considered an early successional stage species^24^, it is suggested that *Cistus* is associated with oak recruitment and with secondary progressive succession^8^. Furthermore, *Cistus* is a multi-hosting species associated with a wide range of ectomycorrhizal (ECM) fungi^25^, which helps not only with the progressive recovery of the vegetation in terms of composition but also with the recovery of the fungal diversity^11,24^. ECM fungi generate barriers against plant pathogens and forage nutrients and water for mycorrhized seedlings in disturbed, abandoned fields^6,16,26,27^. These *Cistus* fields develop into an understory of oaks, commonly known as chaparral, conferring these landscapes with an as yet mostly unexploited potential. Oak forests across the Peninsula are associated with a high level of fungal diversity^28^, which makes them areas of special economic interest in terms of the non-timber forest product resources that they support^29,30^. A negative relationship between oak mortality and ECM richness and diversity has been reported^31^. Following the long-term and intense process of land abandonment that has occurred in Mediterranean areas^32^, attention should be paid to how the successional process develops in these abandoned areas. *C. ladanifer* fields, which are heavily encroaching these unused areas^5^, have recently managed to capture the attention of the forestry research scientists as their perception as economically low-value stands has changed with the realization that these are mycologically prolific productive areas^33,34^. Many ECM species are assumed to be shared by *Cistus* and *Quercus* hosts, despite their unrelatedness in aspects of structure and phylogeny, suggesting that *Cistus* may play a role as a bridge species^29^ and that ECM species play a role in the recruitment of oak seedlings. It has been already seen that the distance from ectomycorrhizal shrubs *Helianthemum bicknellii* of *Quercus macrocarpa* influenced the level of ectomycorrhizal infection of trees by ECM species known to be associated with *H*. *bicknellii* and their ITS RFLP matched with ectomycorrhizal from *Quercus*, supporting the facilitation ability of Cistaceous for the oak recruitment^35^. This aims the effort for study the networking behavior of the fungal-plants relationships as has been stated that there is not a proper compartmentalization among fungal clades regarding the coexistence in hosts and between mycorrhizal types, promoting the resilience and structural stability of natural communities^36,37^.

However, a study has shown that dense scrubland hinders oak recruitment and that it only occurs in open microsites^6^, suggesting that these Mediterranean landscapes need to be managed. Biodiversity can be shifted by the land use through alterations that are generated in the relationships between its components^13^. Therefore, land management could be an interesting tool for the maintenance of a diverse landscape. For example, management practices can induce a nutrient flush, due to organic matter input through clearing, or maintain plant diversity^2^, which also promotes the fungal community. Indeed, leaving some preexisting vegetation undisturbed while implementing a fire-prevention treatment, or when undertaking a reforestation project, could be an appropriate management tool for maintaining or boosting fungal diversity and creating a more resilient ecosystem^38^.

We hypothesize that the transference of the fungal community along the vegetational succession has a facilitative effect, whereby the pre-established mycorrhizal network of the oldest, most differentiated *Cistus* plants enables *Quercus* seedlings to establish by assisting in the fulfilment of their nutritional demands. We suggest that a mycorrhizal networking scenario is established, connecting the different successional vegetation, which enables a mycorrhizal bridge to be formed by ECM species. To verify this hypothesis, we genetically identified and analyzed the soil fungal communities in five different types of stands with two different dominant plants, *Cistus ladanifer* and *Quercus pyrenaica*, which typically form part of the vegetation successional process in western Iberian forests over the Mediterranean basin^29^. The stands were selected by stand age, ranging from a young *Cistus*-type to an intermediate *Quercus*-type stand, to analyze any differences along a successional chronosequence. Soil samples were also analyzed to monitor any shifts occurring in the chemical composition throughout the succession. Thus, our objectives were to evaluate the total fungal richness and the ECM fungal richness, as well as community distribution dynamics, including network analyses of species within forest types at varying successional stages of the two hosts *Quercus* and *Cistus*, and ultimately assess the age-related effects on the fungal community, to obtain a deeper insight into the ongoing ecological processes. Regarding the network analysis, centrality measures (density, centrality degree, betweenness and closeness) were calculated in order to find species with a principal role within the forest system and the successional process.

## Results

### Host vs. forest-type richness

**Figure 1:**
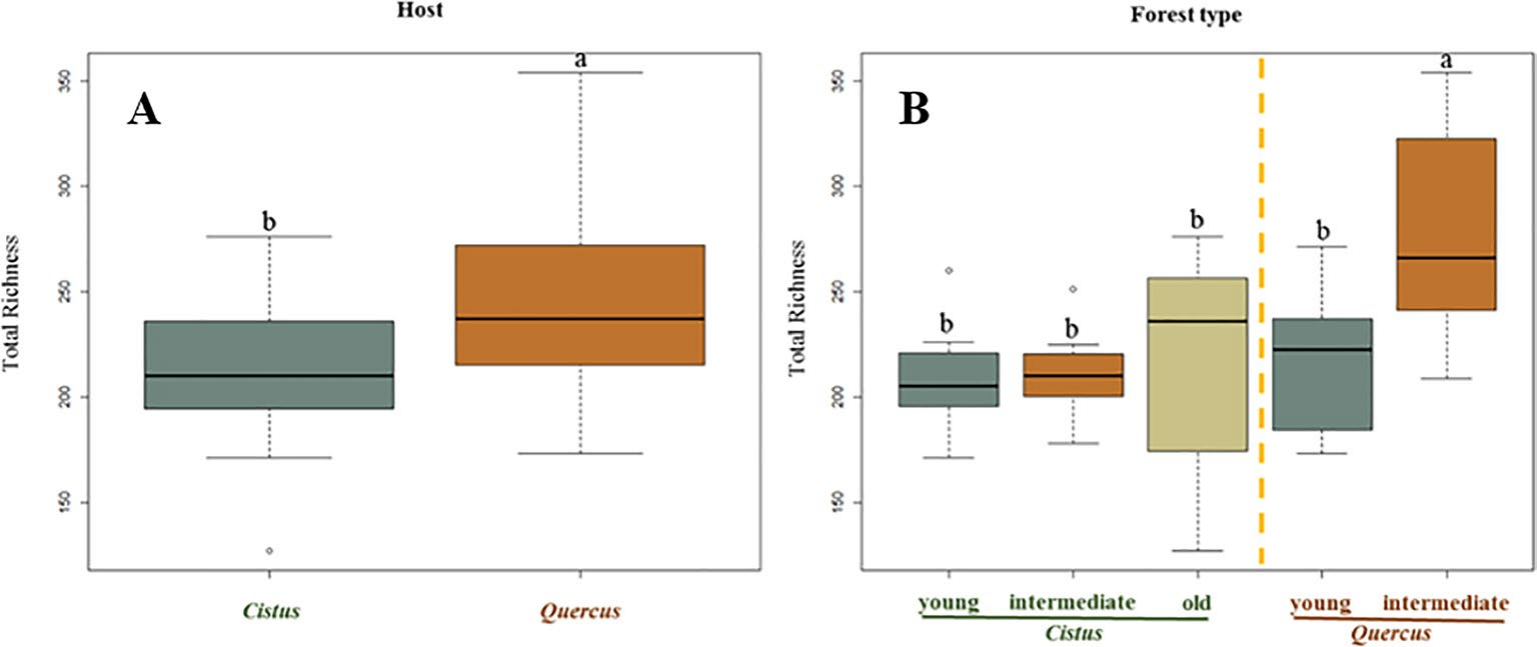
Total fungal operational taxonomic unit richness according to (**A**) host and (**B**) forest types, gathered by the representative forest type species. Different lowercase letters indicate significant differences based on linear mixed effects models and Tukey's HSD test.

The total dataset of sequencing reads was quality-filtered and the LME analysis revealed significant differences in terms of the total richness of the soil fungal communities between *Cistus* and *Quercus* stands but also between the type of forest structure within *Quercus* stands. With regard to total richness, the host role was significant (*P* = 0.007), as was the type of forest (*P* < 0.001). Overall, the soil fungal communities regarding the host showed that the ones associated to *Quercus* were significantly richer than those to *Cistus* (Fig. 1A). The Tukey test showed that the total richness of the most developed *Quercus* forest (intermediate) was significantly higher than that of the *Cistus* stands and the *Quercus* young stand with a *P*-value of < 0.01 (Fig. 1B). By contrast, the total fungal richness of *Quercus* young stands and *Cistus* stands were not significantly different (*P* = 1).

**Figure 2:**
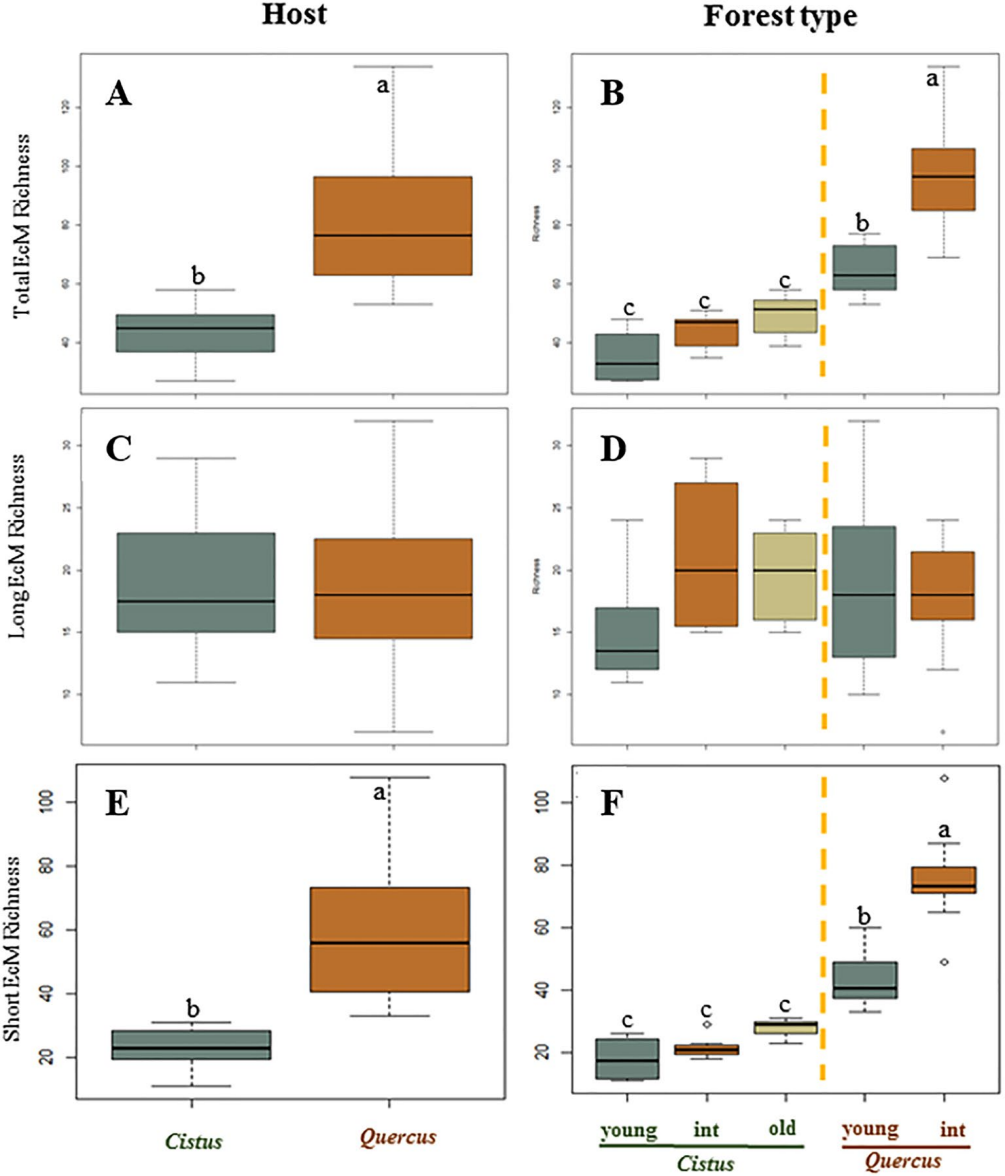
Ectomycorrhizal (ECM) operational taxonomic unit richness according to long- and short-exploration-type distance categories grouped by host and forest types. Different lowercase letters indicate significant differences based on linear mixed effects models and Tukey's HSD test.

In terms of ECM fungi, total richness and the richness of contact/short-distance/medium-distance smooth with hydrophilic hyphae (C/SD/MDS) exploration types were significantly influenced by the host and by the type of forest (*P* < 0.001), with ECM communities and C/SD/MDS exploration types in *Quercus* stands showing significantly higher levels of richness than those in *Cistus* stands (Fig. 2A,B). The Tukey test also revealed that the richness levels of ECM fungi and of C/SD/MDS exploration types in *Quercus* intermediate stands were significantly higher than those of *Cistus* stands and of the *Quercus* young stand (*P* ≤ 0.001). Furthermore, the richness levels of ECM fungi and of C/SD/MDS exploration types in *Quercus* young stands were significantly higher than those of the *Cistus* young, intermediate and old stands (*P* ≤ 0.001) (Fig. 2B,D). However, the richness of medium-distance mat/medium-distance fringe/long-distance with hydrophobic hyphae (MDM/MDF/LD) exploration types did not differ significantly between hosts (*P* = 0.916) or stand structure types (*P* = 0.211) (Fig. 2C,D).

### Effect of host vs. stand structure on the fungal composition

**Figure 3:**
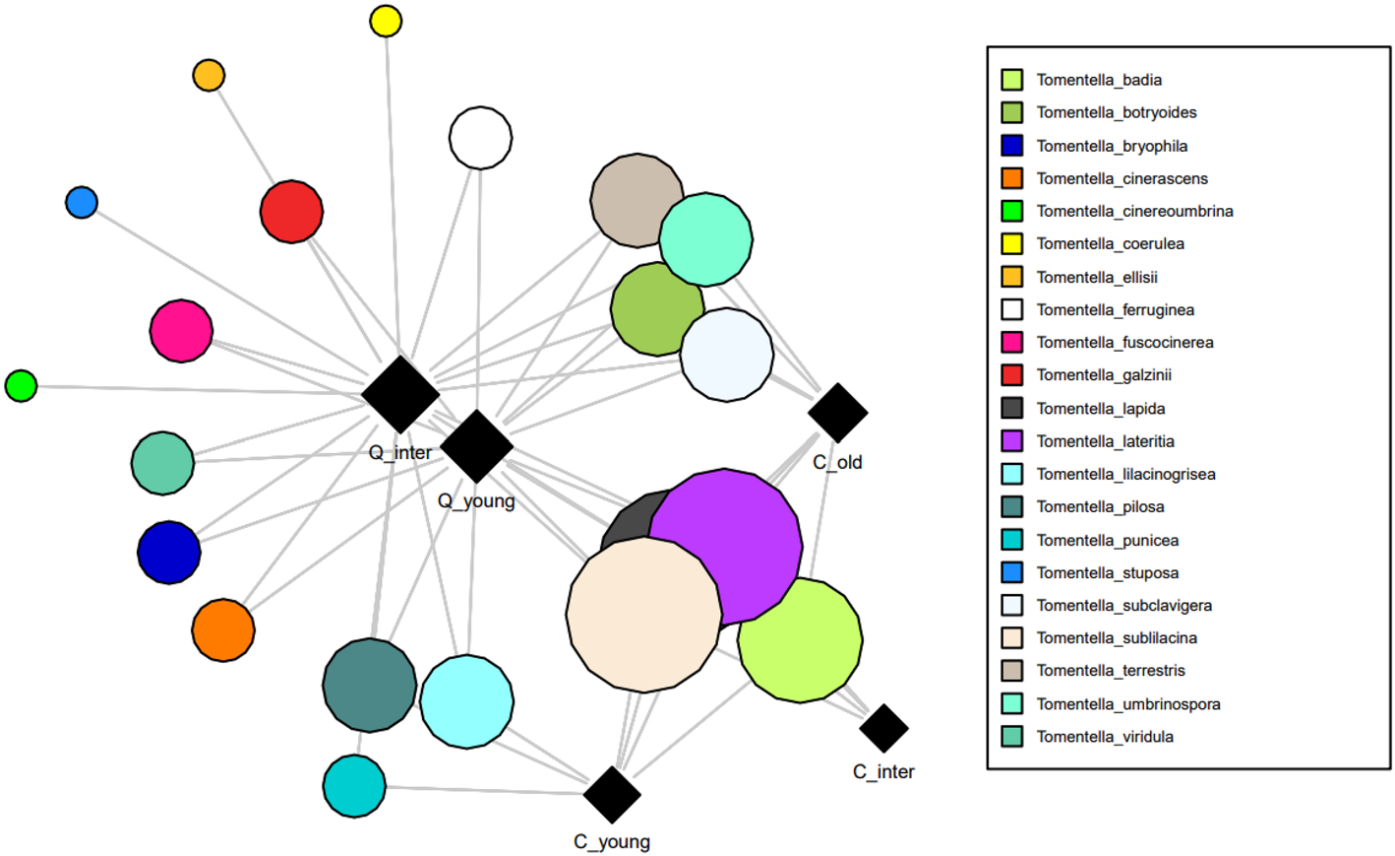
Distribution of operational taxonomic units (OTUs) of taxa belonging to the *Tomentella* genus as visualized by network analysis for the five forest types. Squares and circles indicate sampled forest types and OTUs, respectively. Circles representing OTUs are connected to the forest types (squares) in which they occur by straight lines. Centrally located OTUs were detected in multiple treatments and/or forest types. Circle size is proportional to the number of samples (in the respective forest type) in which the OTU was detected. Network analyses for other relevant genera can be found in the Supplementary Figs. S1–S5. Forest types: *C_old* *Cistus* old, *C_inter* *Cistus* intermediate, *C_young* *Cistus* young, *Q_inter* *Quercus* intermediate, *Q_young* *Quercus* young.

Seven genera were selected for network analysis to visualize the number of times they were detected in the soil of each type of host stand (Fig. 3 and Supplementary Figs. S1–S5). These genera were selected for analysis based on their occurrence in all forest types and comprised more than ten species within each of the *Quercus* Forest stand types. The density calculated in order to obtaining the degree of connectance through the different host type within each genus. *Boletus* (0.25), *Lactarius* (0.21), *Tomentella* (0.17) and *Amanita* (0.12) showed higher values of density indicating that its species showed a higher connectance with all the host types. However, *Cortinarius* (0.065), *Inocybe* (0.049) and *Russula* (0.046) showed a lower connectance in comparison, but gathered a larger number of species dispersing the image of those highly connected species. Centrality indices (degree, betweenness and closeness) showed the relative importance of the different species within each genus in the Net (Table 1), supporting the network plots where the size of each symbol and its proximity to each host type graphically reflects the associations to different forest type (larger the circle, more forest types it is associated with) of each species. In the case of the genus *Amanita*, *Amanita pantherina* was detected in soil at most of the sites, making it the most generalist species within this genus, with the higher values of centrality*.* Besides, *Amanita torrendii* was detected in soils of *Cistus* old and *Quercus* young stand also exhibiting high values of centrality. Among *Russula*, the species *Russula atropurpurea* was detected in more forest types than the other *Russula* species as it was the only species detected in soils of both *Cistus* and *Quercus* stands, also supported by its centrality values. The three species of *Tomentella* that were detected in all forest types and that had the highest number of sequence reads were *Tomentella lapida*,* T. sublilacina* and *T. lateritia* (Fig. 3)*,* being the three of them the ones that gathered the higher values of centrality. Among *Lactarius*, *Lactarius decipiens* and *L. serifluus* were the dominant species in all stand types. The ECM genus with the greatest species richness (69 species) was *Cortinarius*. *Cortinarius obtusus*, *C. purpurascens, C. ohlone* and *C. diasemospermus* were detected in most forest types, especially *Cistus* old and *Quercus* young forest types, and presented the higher values of a centralitya within the genus*.* Finally, network plotting and centrality measures of *Inocybe* revealed that the most shared and central species among forest types were *Inocybe rufoalba, Inocybe rimosa* and *Inocybe krieglsteineri*. The *Quercus* young type showed a higher successional-related proximity to all three *Cistus* types than the *Quercus* intermediate, especially among these central genera. However, a comparably stronger relationship was observed between the *Quercus* intermediate type and *Cistus* old stands than for the other stand types.

**Table 1:** Centrality indices of the analysis networks for the main species observed for the genera with higher presence through all the forest types.

Fungal species	Centrality indices		
	Degree	Betweenness	Closeness
*Lactarius decipiens*	10	38.20	0.043
*Lactarius serifluus*	10	38.20	0.043
*Amanita pantherina*	10	136.37	0.026
*Amanita torrendii*	8	29.48	0.020
*Boletus edulis*	8	15.00	0,166
*Inocybe rimosa*	6	214.12	0.014
*Inocybe krieglsteineri*	8	71.50	0.010
*Inocybe rufoalba*	8	71.50	0.010
*Russula atropurpurea*	6	771.29	0.010
*Tomentella lapida*	10	24.53	0.022
*Tomentella lateritia*	10	24.53	0.022
*Tomentella subilacina*	10	24.53	0.022
*Cortinarius diamospermus*	10	66.64	0.007
*Cortinarius ohlone*	10	66.64	0.007
*Cortinarius purpurascens*	10	66.64	0.007
*Cortinarius obtusus*	8	35.31	0.006

**Figure 4:**
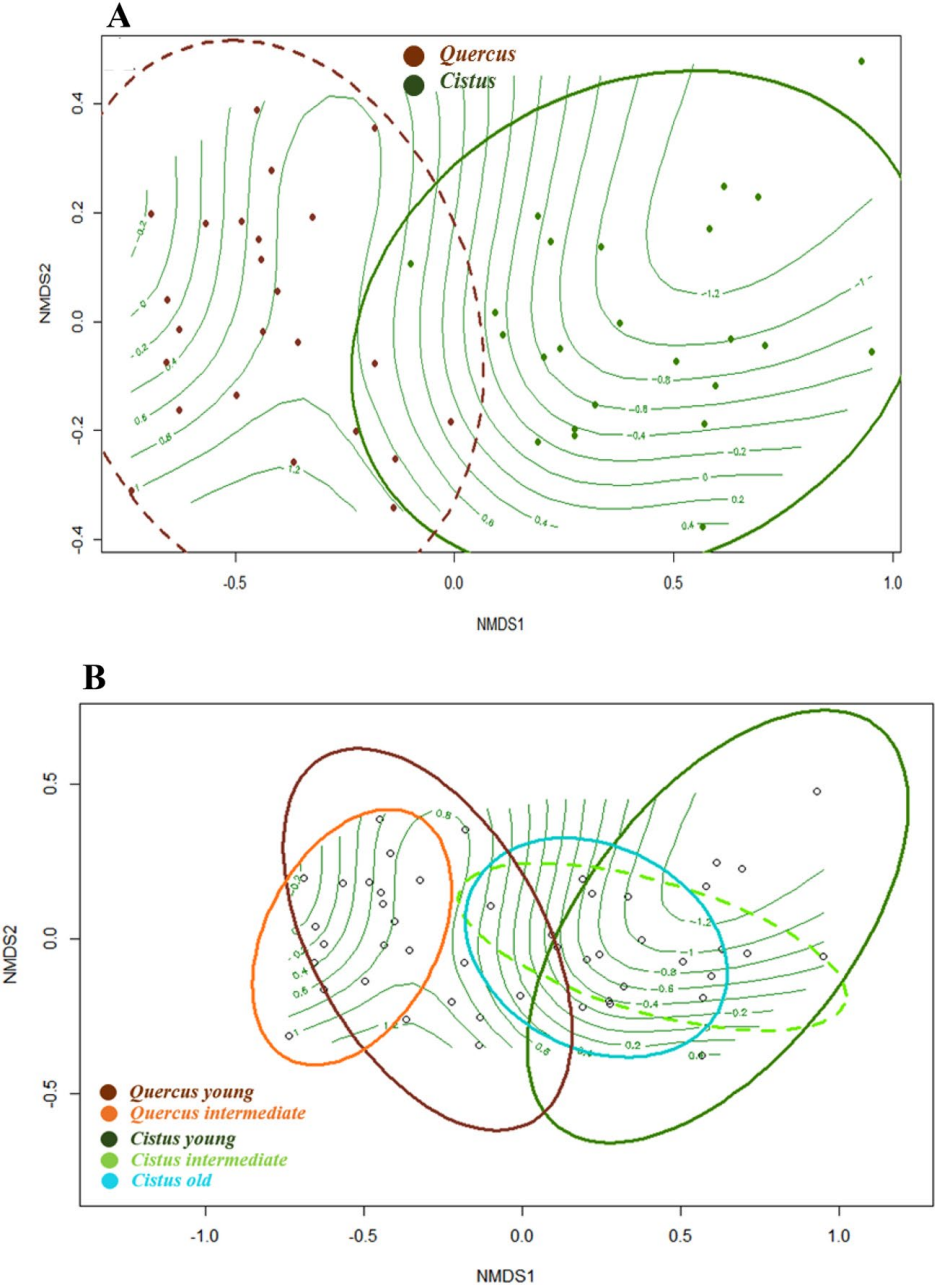
Non-metric multidimensional scaling (NMDS) ordination for ectomycorrhizal (ECM) genera according to (**A**) host and (**B**) forest types. Isolines of the gradient of the C content were also plotted on the NMDS ordinations using the *ordisurf* Model Gaussian function (degrees of freedom, 8.12; REML score, 51.774).

Non-metric multidimensional scaling (NMDS) PerMANOVA test showed that ECM composition was clearly affected by the host species (Fig. 4A) present in the stand (*P* = 0.001; *r*^2^ = 0.415), with a stress for the model of 0.125. The mean significant soil variables affecting the influence of the host over the composition were P (*P* = 0.020), N (*P* = 0.001), C (*P* = 0.001), C:N ratio (*P* = 0.020), and dry matter (*P* = 0.001), and precipitation and temperature (*P* = 0.001) (Table 2). The isolines in Fig. 4A represent the gradient of the C content. There was an overlap of genera shared by both types of hosts, which showed that a high number of *Quercus*-associated genera were harbored by *Cistus* too. Another PerMANOVA analysis also showed that forest type had a significant effect (*P* = 0.001; *r*^2^ = 0.528) on the ECM fungal community and was manifested through a NMDS (Fig. 4B). Isolines in Fig. 4B also represent the gradient of the C. ECM communities in *Cistus* stands were characterized by higher C:N ratios, which influenced the *Cistus* old and *Cistus* intermediate ECM communities, and dry matter, which influenced the *Cistus* young ECM community. By contrast, ECM communities in *Quercus* stands were influenced by pH, and P and N content. The composition of ECM genera in *Quercus* intermediate stands was different to those in *Cistus* stands. However, *Quercus* young stands showed some overlap with *Cistus* old and *Cistus* intermediate stands.

**Table 2:** Significance of explanatory variables for soil fungal community composition based on a Hellinger transformed matrix.

Group	Variables	*r*^2^	*P*
Soil fertility	pH	0.0436	0.368
	P	0.1758	0.020
	N	0.6031	**0.001**
	C	0.4487	**0.001**
	C:N	0.1562	0.020
	Dry matter	0.7152	**0.001**
Climate	Precipitation	0.5002	**0.001**
	Temperature	0.3715	**0.001**

*Numbers in bold indicate a highly significant effect (*P* < 0.01).

## Discussion

### Host and forest type determine fungal, ECM and exploration-type richness

The composition of the soil fungal communities in stands of *Quercus* and *Cistus* differed significantly, giving rise to the assumption that despite the number of shared genera associated with both hosts^25^, there could be some differences in the composition of their fungal communities. *Quercus* stands showed higher levels of richness, indicating that the stage of their development in terms of structure correlated with a later stage of succession than that of *Cistus*, correlating with the larger biodiversity of the ecosystem^39^. This observation was supported by our analysis of the OTU richness of each forest type, which showed that *Quercus* intermediate stands had a higher level of fungal richness than other stands. The model of ECM fungal succession proposed by Peay^40^ describes structural and spatial changes in the root structure, enabling different exploration types to dominate mycorrhization at different times and in different spaces. Common theories of coevolution applied to the complex plant–fungi relationship support this idea, proposing a reciprocal influence on the development of specialized physiological traits of both partners^41^. Furthermore, some studies maintain that older plants with dense root systems harbor a larger ECM community in accordance with a conservative resource acquisition trade-off for plants with slower growth rates^42^. For saprotrophic fungi, although there is a strong correlation between increasing stand age and the richness of the fungal guild^43^, it is also influenced by abiotic variables, such as relative air humidity^44^. However, similarities in species richness between the old *Cistus* stands and the young *Quercus* stands suggest a soil fungal community of shared genera. This could be because of the structural similarity of the understory in young *Quercus* stands and *Cistus* old stands, creating similar environmental conditions in both stand types.

ECM richness showed a similar trend to that observed for total fungal richness. The stand associated with the most differentiated development was *Quercus* intermediate, which hosted the greatest number of ECM genera, as reported by other studies^45^. This could be because a greater number of fine roots are infected with mycorrhiza in older trees^46^, most likely due to the greater quantity of fine root tips typically seen in mid-aged trees compared with young trees^47^. Moreover, ECM richness increased throughout the chronosequence, from the youngest *Cistus* stands to the oldest *Quercus* stands. As expected in Mediterranean forest successions, C content levels increased throughout the vegetational succession, as did the soil N content and the C:N ratio^7,10,32^. High C values in the soil of mycorrhizal regimes correlate with an increase in species richness, which might also support the theory that increased C storage in soils is due to an abundance of ectomycorrhizal fungi, which are able to produce nitrogen-degrading enzymes, causing an accumulation of soil C^48^.

The richness values of the C/SD/MDS exploration types seemed to show a similar trend to those observed for total ECM richness, with the highest richness values recorded for *Quercus* intermediate stands, increasing with stand age, as has been seen in other studies^49^. The high richness values of C/SD/MDS exploration types have been associated with forests with high soil pH and phosphorus content levels, like those in our stands^50^. Furthermore, their limited mobility due to their inability to form rhizomorphic structures or to form rhizomorphs with limited reach, may account for a spatial dependence on the host^51^. Short-distance exploration types of ECM fungi are more closely associated with mature forest than long-distance exploration types^52^, which could be related to moisture hotspots found around nonsuberized root-tips, which favor the hydrophilic mycelium of short-distance exploration types^53^. The higher N content found in the soils of *Quercus* forest types may explain the greater number of short-distance exploration types, which are able to allocate the more labile N compounds to their host trees more easily^54^. We suggest that exploration types that are unable to form rhizomorphic hyphae can fulfill their ecological role by growing out from free soil propagules, thereby supporting seedlings during the successional process until their maturity. By contrast, the richness of MDM/MDF/LD exploration types in the different forest types did not significantly differ, maintaining a virtually constant richness level in soils of all forest types. Although long-distance exploration types are known to be energetically more expensive than short-distance exploration types, these ECM fungi might be able to increase the host plant’s photosynthetic rate by triggering a specific biochemical cascade to meet their high carbon demand^55^. Taking into account the spatial mobility of MDM/MDF/LD exploration types, the development of an extended mycorrhizal net connecting numerous trees could explain the missing influence of stand age on species richness. Combined with their ability to break down complex N compounds and their hydrophobic mycelial behavior^54,56^, MDM/MDF/LD exploration types could be supporting the recruitment of new oak seedlings among the *Cistus* stands.

### Host and forest type determine fungal succession

NMDS to visualize the host’s influence on ECM fungi showed significantly different fungal composition with a distribution mainly driven by the P content and the C:N ratio. An overlap between these two populations seems to occur in soils with increased carbon content. Intermediate *Quercus* stands did not share any genus with *Cistus* stands, whereas the community composition of young oak stands showed some overlap with ECM communities inhabiting soils in the three *Cistus* stands, particularly the *Cistus* old stands. Hence, the colonization of oaks by ECM fungi seems to be facilitated by preestablished *Cistus* mycorrhizal species, supporting similar findings for pines, either due to increased spore and hyphal density or by generating mycorrhizal networks^57^. If this was the case, the development of these interspecies networks was likely facilitating the success of *Quercus* seedlings through the recruitment carried out by mature *Cistus* plants, which could explain why the soil fungal communities of *Quercus* young stands and *Cistus* old stands showed more overlap than those of other forest types. This facilitation of the seedling establishment should happen in a similar way to the process already observed occurring between *Quercus* species (*Q. montana* and *Q. rubra*) fostered by proximity^58,59^. The shade provided by *Cistus* stands may aid the recruitment of *Quercus* seedlings, which are sensitive to high levels of irradiation, and provide them with ECM inoculum after the dry season^15^. Recent studies have even proposed the idea of increased root extension and supportive nutrient exchange between two allospecific tree species if one is exposed to shading^27^, supporting the idea that ECM fungi associated with *Cistus* stands are beneficial for the development of young *Quercus* individuals.

Different vegetation types shape the soil community, normally by changing the abiotic properties of the soil. This could explain the conspicuous difference between the *Quercus* intermediate and the *Cistus* young communities^60^. Early colonizer fungi are likely to colonize *Cistus* stands first, whereas oak-preferring fungi develop compatible mycorrhizal symbioses with rockroses until *Quercus* seedlings are established^61^. Previous studies have shown that nurse shrubs that established earlier in the succession help to facilitate oak recruitment^6^, which could in part be due to the transfer of the *Cistus* fungal community to young *Quercus* individuals. The correlation of the fungal community with a particular forest type depending on the pH level agrees with findings reported by other studies^62,63^, particularly for ECM fungi, as a high pH negatively affects cellulose-degrading enzymes^44^.

Within the *Cistus* old and *Quercus* young stands, there appears to be a shared community that bridges the gap between the early and the later stages of the succession. This shared community and the characteristics of both stands support the idea that mature rockroses help the recruitment of *Quercus* seedlings through mycorrhizal networks^57^. Network plotting revealed those ECM genera that were attached to all forest types, occurring throughout the succession, gathering enough number of species to discern which of them displayed a stronger successional role between forest types. Density measurements gave and idea of the level of connectivity of these genera presented through all the forest succession. It can be seen than among those with a higher density value, *Boletus* was the higher one but just gathered three species in comparison to *Lactarius* that was close to *Boletus* but gathered more species, indicating probably a larger number of links. On the other hand, although *Inocybe*, *Russula* and *Cortinarius* showed a lower density value, all of them gathered considerably more species probably hiding the large connectivity of some species. Furthermore, the centrality indices measured supported the central role^64^ of certain species among those more ubiquitous genera present through all the forest types. The degree indicates that this species gathered more interactions with the different type of forests^65^, indicating the importance of a species^66^, suggesting to being more generalized through the hole succession. The higher betweenness indicates a shorter connection with the forest type node, meaning a mediating role in between hosts types^67^, facilitating bridge connections as mycorrhizal hubs. Finally, the centrality indicated their larger connectivity with the forest types in comparison with the rest of species and a main and closer position to the forest type nodes^65,66^.

*Amanita* is a genus associated with middle aged or mature stands, with some species such as *Amanita muscaria*, acting as an indicator of young trees^68^. This could suggest that this genus plays a role in the transference of ECM mycelium from *Cistus* to *Quercus* stands. Previous studies have shown that the acquisition of P by *Amanita pantherina* could be driven by the enrichment of phosphate-solubilizing bacteria, such as *Pseudomonas fluorescens*, acting as mycorrhizal helper bacteria (MHB)^69,70^. This would also support the idea of seedling recruitment being aided by both enriched MHB and *Amanita* species such as *A. pantherina,* which is supported by it high betweenness values*. Amanita torrendii* has been associated with other Cistaceae members such as *Halimium* sp.^71^ and *Cistus salvifolius*^72^ as well as with *Quercus* stands^73^. The genus *Lactarius* is associated with the late stages of succession, forming associations with a broad spectrum of hosts from woody trees to shrubs^74^. The species detected most frequently in all forest types were *Lactarius decipiens* and *Lactarius serifluus*. *L. decipiens* is specially associated to broad-leaf trees, especially oaks^75^ as can be supported by it centrality degree. *Russula* is a widespread ECM genus that is known to be associated with different hosts of different ages^76–78^, so it is unsurprising that the species *R. atropurpurea* was found in both *Cistus* and *Quercus* young stands. This *Russula* species appeared to be involved in the succession process as it was the only species in this genus that was associated with both hosts, also supported by the centrality measures highlighting its betweenness value. *Tomentella lapida* is found worldwide associated with different types of hosts^79^ and is a strong colonizer that is not affected by C supply disruption^80^. *Tomentella sublilacina* has a broad host range^81^, including bud-bursting trees, areas of afforestation^82,83^ and temperate oak forests^84,85^, which suggests that this species could be involved in the recruitment process of *Quercus* seedlings. Although *Cortinarius* is known to be a highly dominant genus in old forest^86^, especially in oak stands^87,88^, it has also been reported to form a powerful symbiotic relationship with the Cistaceae family^71^. *Cortinarius* species play an important role in stand succession due to their active role in the degradation of lignin and complex organic matter^86^. Therefore, *Cortinarius* could help in the recruitment of oak seedlings, not only as a source of ECM inoculum that could be transferred from rockroses to oaks but also via N fixation in soils with a low N content, such as the soils in our *Cistus* stands^89^. Members of this genus (*Cortinarius obtusus* and *Cortinarius diasemospermus*) have been shown to have peroxidase activity, enabling them to mobilize N^90^. *Cortinarius* spp. have been shown to have an increasing correlation with maturing vegetation and are predictors of tree emergence^91^, which could mean that the species mentioned above are related to the recruitment of seedlings in the succession from *Cistus* fields to *Quercus* forests. *Inocybe*, a well-known ECM genus that is among the first to appear after disturbances^45^, dominates soil fungal communities in holm oak (*Quercus ilex*) stands^87^. The three species of this genus that were detected most frequently in all types of forests were *Inocybe krieglsteineri*, *Inocybe rimosa* and *Inocybe rufoalba.* The observed presence of *I. rufoalba* in five-month-old chestnut seedlings supports this idea^92^, as *I. krieglsteineri* has been associated to different types of *Quercus* species^93,94^.

This study revealed that *Quercus* stand types in general, but particularly the more mature stands, are richer in terms of fungal diversity than *Cistus* types. Furthermore, ECM diversity showed a similar trend, with a noticeable increase in short-distance exploration types with age whereas long-distance exploration types did not vary significantly. Compositional analyses and network analyses revealed that the greatest overlap in terms of ECM fungal community composition during the successional process occurred between old *Cistus* understory and young *Quercus* trees, with *Amanita*, *Cortinarius*, *Lactarius*, *Inocybe*, *Russula*, and *Tomentella* playing a key role as bridging species between the two host species. Attention should be paid to these bridging species to gather more information about their role as *Cistus* fields are replaced by oak forests and their possible engagement in tree recruitment or seedling survival facilitation.

## Materials and methods

### Study area

The study sites were located in the central-west part of Spain, in the supra-Mediterranean thermotype provinces of Leon and Zamora^95^ Paleozoic metamorphic (slate and quartzite) and plutonic (granite) rocks dominates the landscape in addition with tertiary sands. The climate is characterized by two marked seasons of at least two dry months in the summer and a cold winter, with temperate intermediate seasons. The mean annual precipitation is 450–700 mm, with the majority of the precipitation falling between October and February. Mean annual temperature variates from 9.5 to 11.3 °C. Mature intermediate-sized forests are mainly represented by *Quercus pyrenaica*-dominated woodland (Quercion pyrenaicae; Natura 2000 code 9230) and, to a lesser extent, by *Quercus rotundifolia* woodland (Quercion broteroi; Natura 2000 code 9340). In addition, plantations of maritime pine (*Pinus pinaster*) and chestnut (*Castanea sativa*) are relatively common in the study area.

Unmanaged young and coppiced stands of *Q. pyrenaica* is quite extended in the study area presenting a large stem density due to sprouting ability. However, high forest stands with a closed canopy get to developed from coppiced stands receiving silvicultural management during the last 15 years. High forest stands present a cover density between 85 and 100%. All through the coppice stands, shrubs communities dominated by *Cistus ladanifer* and spontaneously *Genista* sp. and *Calluna vulgaris* appears. Furthermore, monospecific shrubby areas are dominated by *Cistus ladanifer* presenting a large density 90–100% cover. Differentiation can be found between the 2 m-tall old Cistus and the 1-m-tall young ones, with intermediate phases.

The studied plot of *Quercus* was divided between a high-density monospecific unmanaged coppice stand (from now *Quercus* young); and a low-density high forest stands managed during the last 15 years (from now *Quercus* intermediate). Regarding *Cistus ladanifer*, three different plots were studied comprising a young *Cistus* field of 4–6 years old, a middle/intermediate sized stand between 10 and 12 years old and finally an old *Cistus* stands of 20–22 years old. Information was obtained from the Regional Forest Management Services and the study was developed in two areas including all the fives forest types. The experimental plots were composed of six plots (50 m × 50 m) of each of the *Quercus* forest types (2 × 6 = 12 plots) in both sites (24 plots) and four plots of each of the *Cistus* forest types (3 × 4 = 12 plots) in both sites (24 plots), making a total of 48 plots (24 *Quercus* and 24 *Cistus* plots).

### Soil sampling and molecular work

Soil sampling was carried out during the 2019 spring with an auger (2 cm radius, 20 cm deep and 250 cm^3^) taking 15 from Cistus plots and 25 from the Quercus plots, and pooling in one sample per plot for each plot type. For the Quercus one, there were randomly selected five trees with an 8 m separation, taking the sample removing the debris from the different sides of the tree at 2 m apart of it base and with another 2 m of separation between them. Afterwards, they were dried a room temperature until a constant weight and sieved (1 mm). For genomic DNA analysis 100 g of each sample were used and two 20 g samples were stored at – 20 °C until the physical–chemical analysis. The physical–chemical analysis was carried out following Sparks et al.^96^ to determine the soil pH (water-based), dry matter (%), total phosphorus (P), total carbon (C), and total nitrogen (N) content of the samples (Table 3).

**Table 3:** Summary statistics for the best linear mixed model fitted to the forest type and host with the fixed effects of edaphic and climatic variables.

Variable	Forest type					Host	
	*Cistus* young	*Cistus* intermediate	*Cistus* old	*Quercus* young	*Quercus* intermediate	*Cistus*	*Quercus*
pH	5.04ac	4.73ab	4.70ab	4.66b	5.33c	4.82a	4.99a
P	10.74a	9.38a	10.83a	8.25a	11.34a	10.30a	9.79a
N	0.11a	0.11a	0.12a	0.26b	0.29b	0.11a	0.28b
C	1.58a	1.70a	1.87a	3.53b	3.33b	1.71a	3.43b
C:N	13.68ac	14.72ab	15.84a	13.36abc	11.60c	14.74a	12.48b
Dry matter	99.25	99.30	99.12	98.13	97.83	99.22a	97.97b
Prec. (mm)	585.50	585.50	585.50	735.00	735.00	585.50	735.00
Temp (°C)	10.40	10.40	10.40	11.50	11.50	10.40	11.50

*Different lowercase letters indicate significant differences among forest types or hosts based on linear mixed effects models and Tukey's HSD test. Precipitation (Prec.) and temperature (Temp.) values are annual means.

DNA extraction was carried out by a Qiagen Powerlyzer-PowerSoil™ DNA Isolation Kit (MoBio Laboratories Inc., Carlsbad, CA, USA) from 0.25 g of soil per sample following it protocol. PCR reactions per sample, carried out in triplicates to minimize the bias, were performed in 20 μl reaction volumes containing 11.22 μl of modified quantization (MQ) water, 1.60 μl of DNA template, 2.00 μl of 10 × buffer, 1.40 μl of MgCl_2_ (50 mM), 1.60 μl of dNTPs (10 mM), 0.50 μl of bovine serum albumin (2%), 0.80 μl of reverse and forward primers (10 μM), and 0.08 μl of Platinum Taq polymerase (Invitrogen, Carlsbad, CA, USA). The ITS2 region (ca. 250 bp) of the nuclear ribosomal DNA repeat was PCR-amplified using primers fITS7^97^ and ITS4^98^. Sample-specific Multiplex Identification DNA-tags were used to label the ITS4 primer. Negative controls containing MQ water were included. Illimina MiSeq platform by BaseClear B.V. company (Leiden, The Netherlands) was used for sequencing.

### Bioinformatic analysis and statistical analysis

Cutadapt^99^ for trimming low-quality ends and merging paired read through USEARCH v.10.0.240^100^. It was set up with a quality score of 5 and a 200 bp length minimum sequence. ITS4 and fITS7 were trimmed and sequence with an expected error of > 1 were removed. Remaining sequences were merged and high-quality ones were grouped at 97% similarity through USEARCH into a map of operational taxonomic units (OTUs) representing OTUs with > 70% similarity or > 200 bp pairwise alignment length to a fungal sequence.

Assignment to taxonomic groups by pairwise similarity against the UNITE fungal ITS sequence database^101^, containing identified fungal sequences with assignments to Species Hypothesis (SH)^102^, through PlutoF web workbench (https://plutof.ut.ee)^103^. If a with > 90% similarity to a fungal SH was obtained, OTUs were assigned to functional group^104^. ECM fungao OTUs were classified into two aggregate mycelial exploration-type categories: contact/short-distance/medium-distance smooth with hydrophilic hyphae (C/SD/MDS) and medium-distance mat/medium-distance fringe/long-distance with hydrophobic hyphae (MDM/MDF/LD) following^105,106^, and the DEEMY database (http://deemy.de) (Table 4).

**Table 4:** Number of detections of ectomycorrhizal (ECM) genera detected in soils of different forest types and grouped by host.

ECM genera	ECM exploration type	Forest type					Host	
		*Cistus* young	*Cistus* intermediate	*Cistus* old	*Quercus* young	*Quercus* intermediate	*Quercus*	*Cistus*
*Amanita*	MDM/MDF/LD	8	8	8	11	12	23	24
*Astraeus*	MDM/MDF/LD	4	3	2	0	0	0	9
*Bankera*	MDM/MDF/LD	0	0	1	3	0	3	1
*Boletopsis*	MDM/MDF/LD	0	0	0	2	0	2	0
*Boletus*	MDM/MDF/LD	3	8	7	5	1	6	18
*Cantharellus*	C/SD/MDS	0	1	0	6	7	13	1
*Cenococcum*	C/SD/MDS	8	6	8	12	12	24	22
*Choiromyces*	C/SD/MDS	0	1	0	3	0	3	1
*Clavulina*	C/SD/MDS	1	0	4	6	8	14	5
*Cortinarius*	MDM/MDF/LD	8	8	8	12	12	24	24
*Craterellus*	C/SD/MDS	0	0	0	0	3	3	0
*Elaphomyces*	C/SD/MDS	0	0	0	8	12	20	0
*Geopora*	C/SD/MDS	8	8	8	7	0	7	24
*Gymnomyces*	C/SD/MDS	0	0	0	0	3	3	0
*Gyroporus*	MDM/MDF/LD	1	0	0	2	4	6	1
*Helvellosebacina*	C/SD/MDS	0	0	0	0	7	7	0
*Humaria*	C/SD/MDS	0	0	0	1	9	10	0
*Hydnellum*	MDM/MDF/LD	0	0	1	5	1	6	1
*Hydnobolites*	C/SD/MDS	0	0	0	0	4	4	0
*Hydnotrya*	C/SD/MDS	0	1	0	0	4	4	1
*Hygrophorus*	C/SD/MDS	3	1	2	2	6	8	6
*Hymenogaster*	C/SD/MDS	0	0	0	1	2	3	0
*Inocybe*	C/SD/MDS	3	8	8	12	12	24	19
*Laccaria*	C/SD/MDS	7	7	7	12	12	24	21
*Lactarius*	C/SD/MDS	8	7	8	10	12	22	23
*Lactifluus*	C/SD/MDS	0	0	0	8	12	20	0
*Lyophyllum*	MDM/MDF/LD	1	0	1	2	0	2	2
*Membranomyces*	C/SD/MDS	0	0	0	0	4	4	0
*Otidea*	C/SD/MDS	0	0	0	1	5	6	0
*Pachyphloeus*	C/SD/MDS	1	0	0	0	3	3	1
*Paxillus*	MDM/MDF/LD	1	0	0	0	0	0	1
*Phellodon*	MDM/MDF/LD	0	0	0	1	0	1	0
*Pisolithus*	MDM/MDF/LD	5	2	1	0	1	1	8
*Pseudocraterellus*	C/SD/MDS	0	0	0	6	0	6	0
*Pseudotomentella*	C/SD/MDS	0	0	0	0	2	2	0
*Rhizopogon*	MDM/MDF/LD	4	4	4	0	1	1	12
*Russula*	C/SD/MDS	5	7	8	12	12	24	20
*Scleroderma*	MDM/MDF/LD	4	2	0	3	6	9	6
*Sebacina*	C/SD/MDS	3	3	1	11	12	23	7
*Tarzetta*	C/SD/MDS	0	0	0	0	7	7	0
*Terfezia*	C/SD/MDS	8	8	8	6	2	8	24
*Thelephora*	C/SD/MDS	5	8	8	9	9	18	21
*Tomentella*	C/SD/MDS	7	8	8	12	12	24	23
*Tricholoma*	MDM/MDF/LD	0	1	4	8	12	20	5
*Tuber*	C/SD/MDS	6	1	4	12	9	21	11
*Xerocomellus*	MDM/MDF/LD	0	0	0	5	1	6	0
*Xerocomus*	MDM/MDF/LD	0	2	0	7	7	14	2

Data used for statistical analyses were transformed when needed to achieve the parametric criteria of normality and homoscedasticity. Normalization by rarefying the abundance data to the smallest number of OTUs per plot. In addition, soil variable data were scaled using base R, richness values for each forest were also estimated and, finally, diversity measures were analyzed using the Biodiversity R package^107^ in R version 4.0.3^108^.

For assessing difference between forests plots Linear Mixed Effects (LME) models^109^ were used defining the plots in each forest area and the forest type as the random and fixed factors, respectively. For testing significant differences at a 95% of signification, Tukey test were used.

Non-metric Multidimensional Scaling (NMDS) based on a Hellinger-transformed OTU matrix for ECM genera and environmental scaled data for represent fungal composition and edaphic and forests types relationship. *Ordisurf* function was used to plot isolines of vascular plant richness A Permutational Multivariate ANOVA (PerMANOVA) based on 999 permutations using the *adonis* function in the vegan package was carried out to assess the forest type effect. *Ordisurf* function was used to plot isolines of vascular plant richness. Edaphic variables influence over the fungal composition was based on a Bray–Curtis dissimilarity matrix, excluding singleton OTUs, and correlation of NMDS axes with explanatory variables were assessed through *envfit* R function. A network display to visualize the fungal OTUs distribution among the forest types at species level of the more ubiquitous fungal genera was carried put using the R package *sna*^110^.

To describe the network associations, the density of each net was calculated using *dgen* function. Then, to evaluate the host association of each fungal OUT within each net, we scored fungal OTUs based on their topological positions through the calculation of centrality indices to interpret their role within the different forest hosts and stages, using *igraph* package in R. Different centrality indices were calculated. Thus, *Density* is the fraction of actual links in relation to the total number of possible links^111^, *Degree* shows the number of edges that connect the focal node to other nodes, *Betweenness* notes the number of shortest paths that the focal node lies on and *Closeness* the mean shortest path between a focal node and all other nodes in the network^64^. These indices were calculated using *degree, closeness* and *betweenness* functions over our graph object from each studied data frame. Finally, the networks were visualized using *gplot* in R. It was used to produce a two-dimensional plot, where “twomode” was selected as gmod. Plotting concentric circles based on the magnitude of a covariate it has been carried out using *gplot*.*target* that supplies a front-end to *gplot*. Those OTUs not assigned to species level were excluded of the network representation.

### Vegetal material sampling permit statement

Specific permits were not needed as sampling of plants was not carried out in this research.

### Methods statement

All methods were carried out in accordance with relevant guidelines and regulations.

## Supplementary Information

Supplementary Figures.

## Data Availability

Submission number GenBank: SUB12065066.
