# Supplementary material for: Into the void: ECM fungal communities involved in the succession from rockroses to oak stands
Source: Sci Rep. 2023 Jun 21;13:10085. doi: 10.1038/s41598-023-37107-y (PMC10284853; doi:10.1038/s41598-023-37107-y)
Supplement: Supplementary file 1 — Supplementary Figures. [file 41598_2023_37107_MOESM1_ESM.docx]

Title: Into the void: ECM fungal communities involved in the succession from rockroses to oak stands

Ignacio Sanz-Benito1, Tim Stadler2, Olaya Mediavilla1,3, María Hernández-Rodríguez1,3, Juan Andrés Oria-de-Rueda1, Tatek Dejene1,4, József Geml5, Pablo Martín-Pinto1,*

Address:

1Sustainable Forest Management Research Institute, University of Valladolid, Avda. Madrid 44, 34071, Palencia, Spain

2University for Sustainable Development Eberswalde, Schickler Street 5, 16225, Eberswalde, Germany

3IDForest-Biotecnología Forestal Aplicada, Calle Curtidores 17, 34004, Palencia, Spain

4Central Ethiopia Environment and Forestry Research Center, P.O. Box 30708, Addis Ababa, Ethiopia

5MTA-EKE Lendület Environmental Microbiome Research Group, Eszterházy Károly University, Leányka u. 6, 3300 Eger, Hungary

*Corresponding author:

Pablo Martín-Pinto

Phone.+34979108340 Fax. +34979108440

e-mail. [pmpinto@pvs.uva.es](mailto:pmpinto@pvs.uva.es)

*Lactarius*


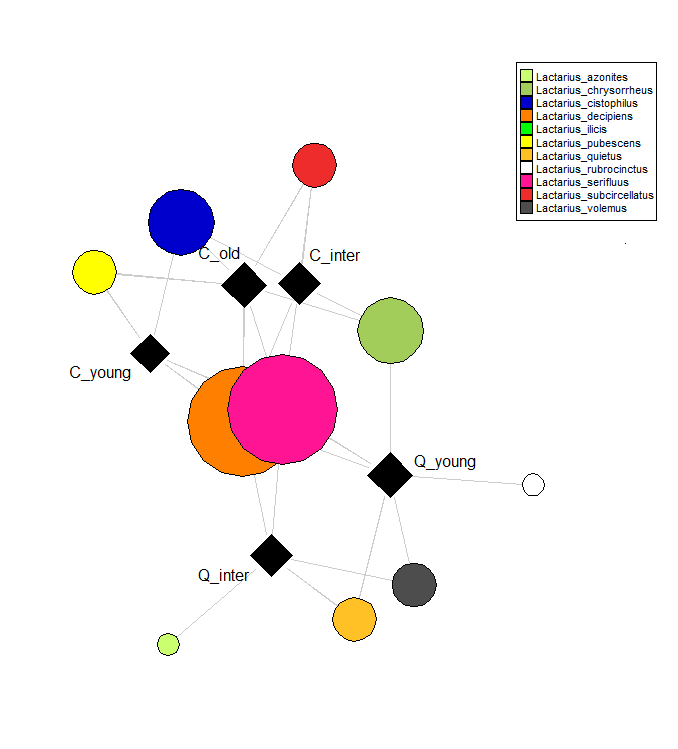


**Figure S1** Distribution of operational taxonomic units (OTUs) of taxa belonging to the *Lactarius* genus as visualized by network analysis for the five forest types. Squares and circles indicate sampled forest types and OTUs, respectively. Circles representing OTUs are connected to the forest types (squares) in which they occur by straight lines. Centrally located OTUs were detected in multiple treatments and/or forest types. Circle size is proportional to the number of samples (in the respective forest type) in which the OTU was detected. Network analyses for other relevant genera can be found in the supplementary material. Forest types: C_old, *Cistus* old; C_inter, *Cistus* intermediate; C_young, *Cistus* young; Q_inter, *Quercus* intermediate; Q_young, *Quercus* young

*Amanita*


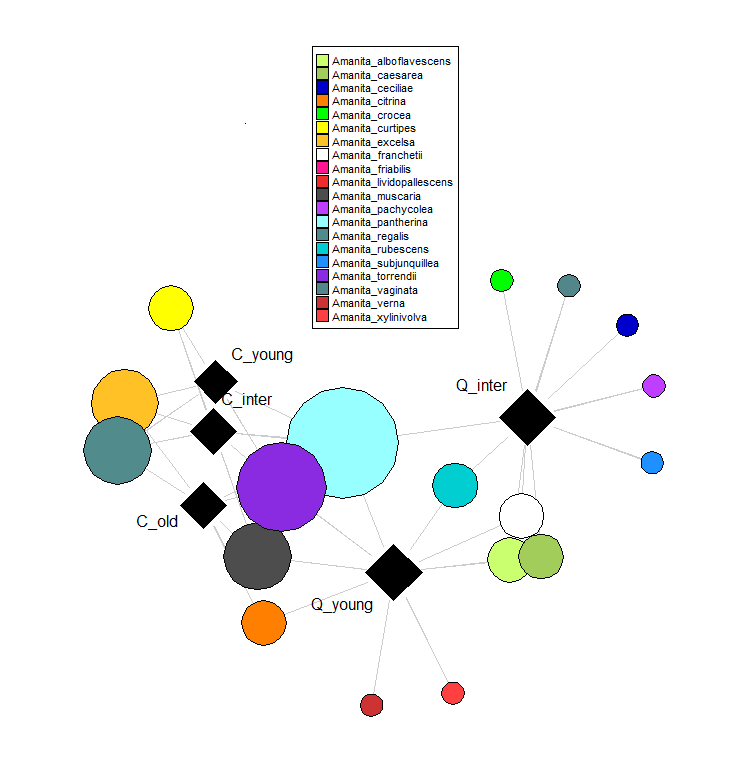


**Figure S2** Distribution of operational taxonomic units (OTUs) of taxa belonging to the *Amanita* genus as visualized by network analysis for the five forest types. Squares and circles indicate sampled forest types and OTUs, respectively. Circles representing OTUs are connected to the forest types (squares) in which they occur by straight lines. Centrally located OTUs were detected in multiple treatments and/or forest types. Circle size is proportional to the number of samples (in the respective forest type) in which the OTU was detected. Network analyses for other relevant genera can be found in the supplementary material. Forest types: C_old, *Cistus* old; C_inter, *Cistus* intermediate; C_young, *Cistus* young; Q_inter, *Quercus* intermediate; Q_young, *Quercus* young

*Inocybe*


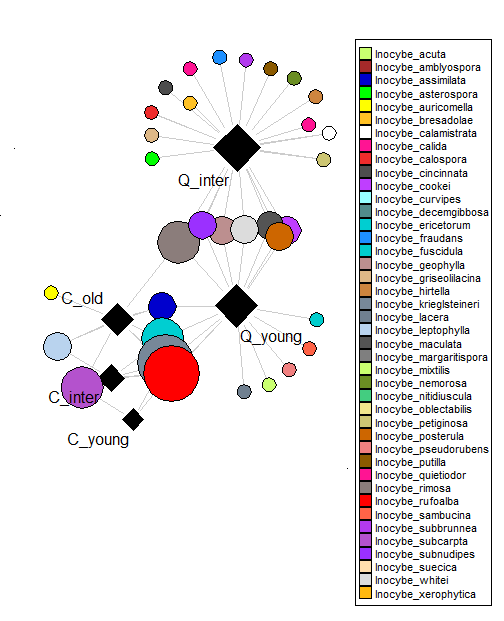


**Figure S3** Distribution of operational taxonomic units (OTUs) of taxa belonging to the *Inocybe* genus as visualized by network analysis for the five forest types. Squares and circles indicate sampled forest types and OTUs, respectively. Circles representing OTUs are connected to the forest types (squares) in which they occur by straight lines. Centrally located OTUs were detected in multiple treatments and/or forest types. Circle size is proportional to the number of samples (in the respective forest type) in which the OTU was detected. Network analyses for other relevant genera can be found in the supplementary material. Forest types: C_old, *Cistus* old; C_inter, *Cistus* intermediate; C_young, *Cistus* young; Q_inter, *Quercus* intermediate; Q_young, *Quercus* young

*Cortinarius*


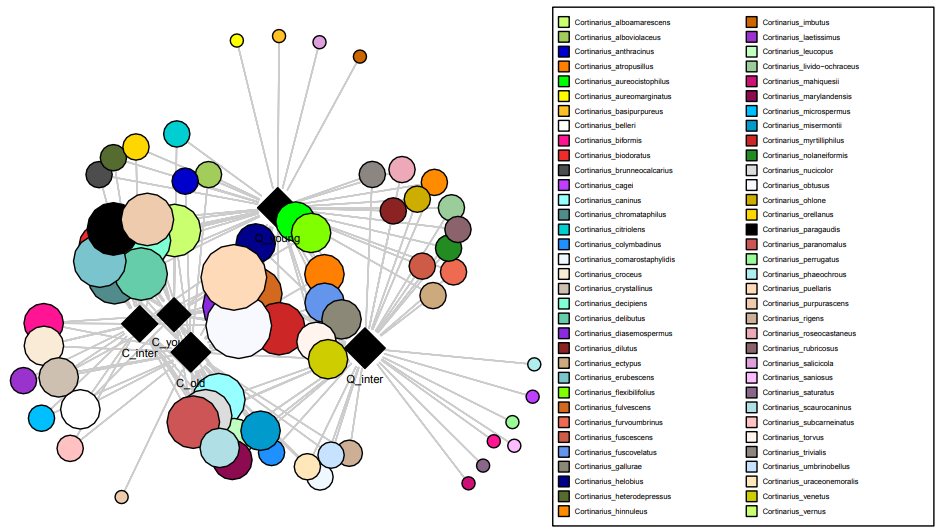


**Figure S4** Distribution of operational taxonomic units (OTUs) of taxa belonging to the *Cortinarius* genus as visualized by network analysis for the five forest types. Squares and circles indicate sampled forest types and OTUs, respectively. Circles representing OTUs are connected to the forest types (squares) in which they occur by straight lines. Centrally located OTUs were detected in multiple treatments and/or forest types. Circle size is proportional to the number of samples (in the respective forest type) in which the OTU was detected. Network analyses for other relevant genera can be found in the supplementary material. Forest types: C_old, *Cistus* old; C_inter, *Cistus* intermediate; C_young, *Cistus* young; Q_inter, *Quercus* intermediate; Q_young, *Quercus* young

*Russula*


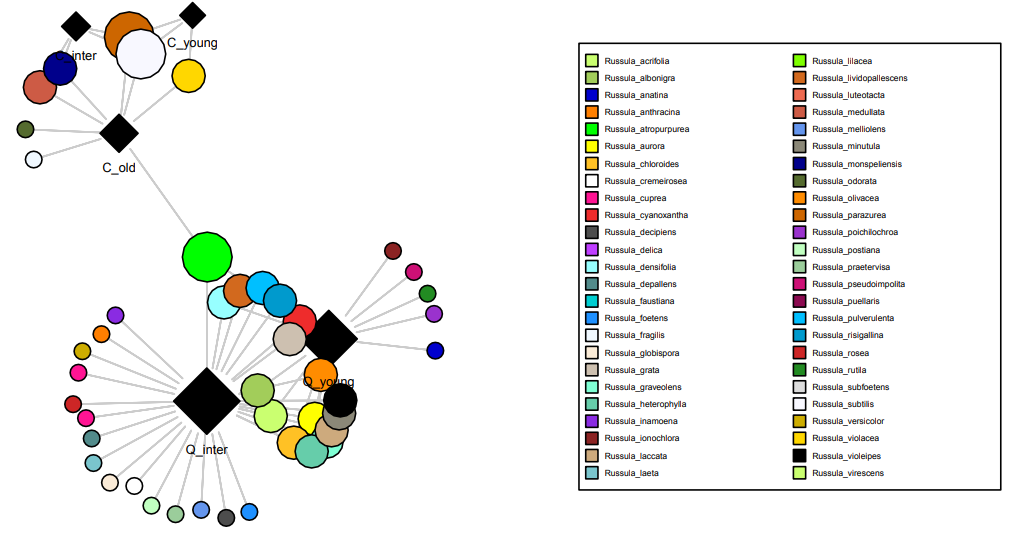


**Figure S5.** Distribution of operational taxonomic units (OTUs) of taxa belonging to the *Russula* genus as visualized by network analysis for the five forest types. Squares and circles indicate sampled forest types and OTUs, respectively. Circles representing OTUs are connected to the forest types (squares) in which they occur by straight lines. Centrally located OTUs were detected in multiple treatments and/or forest types. Circle size is proportional to the number of samples (in the respective forest type) in which the OTU was detected. Network analyses for other relevant genera can be found in the supplementary material. Forest types: C_old, *Cistus* old; C_inter, *Cistus* intermediate; C_young, *Cistus* young; Q_inter, *Quercus* intermediate; Q_young, *Quercus* young.
